# Filling Fraction and Single Beat Method for Estimating Dead-Space Volume of Left Ventricle

**DOI:** 10.1007/s13239-025-00809-7

**Published:** 2026-03-02

**Authors:** Melanie P. Hager, George V. Letsou, John C. Criscione

**Affiliations:** 1 Department of Biomedical Engineering, Texas A&M University, 5045 Emerging Technologies Building, Office 1041J, 3120 TAMU, 101 Bizzell Street, College Station, TX 77843 USA; 2 College of Medicine, Texas A&M University, 8447 Riverside Parkway, Bryan, TX 77807 USA; 3 College of Medicine, University of Houston, 5055 Medical Circle, Houston, TX 77204 USA; 4 TransMedics, Inc, Andover, MA 01810 USA

**Keywords:** Preload, Diastolic Dysfunction, Ejection Fraction, Systolic Dysfunction, HFpEF, HFrEF, Contractility

## Abstract

Accurate assessment of cardiac preload remains a clinical challenge, particularly in heart failure patients where preload manipulation is impractical or contraindicated. Ejection fraction (EF), while widely used, reflects a combination of preload, contractility, and afterload, and may obscure the specific contribution of preload. Inspired by a preload recruitable stroke work (PRSW) model for heart function, a concise, three factor expression for ejection fraction (EF) can be obtained where EF is directly proportional to preload, directly proportional to contractility, and inversely proportional to afterload. The contractility and afterload factors are common metrics; whereas the preload factor, filling fraction (FF), is a novel, dimensionless preload metric that sets a physiologic upperbound on EF. Unfortunately, FF measurement requires an estimate of the volume intercept or dead-space volume of the left ventricle, and such a measure has, conventionally, required preload manipulation with extrapolation to the volume axis. To address this, we propose a single-beat extrapolation method for estimating the volume intercept using end-diastolic and end-systolic measurements. In-silico testing across 10,000 synthetic left ventricular geometries demonstrates strong agreement between the estimated intercept and actual, prescribed dead-space volumes. This method enables non-invasive computation of FF from single-beat data without the need for in vivo preload manipulation. Potential clinical applications include characterizing cardiac function, stratifying heart failure subtypes, and enhancing diagnostic precision when EF is preserved but preload reserve is impaired. Future validation in clinical imaging datasets is warranted to assess real-world applicability.

## Introduction

Ejection fraction (EF) is a critical clinical parameter for characterization of heart function. Heart failure with preserved EF (HFpEF) is defined by EF > 0.5. Heart failure with reduced EF (HFrEF) is defined by EF < 0.5. To gain further insight into EF, we used a preload recruitable stroke-work (PRSW) model of heart function to obtain, perhaps, the most concise representation for EF possible. Specifically, with the definition of a new preload metric, filling fraction (FF), it follows that EF can be expressed in terms of 3 separable factors that individually represent preload, contractility, and afterload, i.e. the classic triad of cardiac output (CO). Moreover, we demonstrate that changes in FF, that are typical with heart dilation, should result in EF < 0.5 even if the contractility does not change. Thus, this model introduces FF as a preload-specific constraint on EF capable of offering insight into EF reductions due to preload, not contractility. FF may enables phenotyping of heart failure patients, especially in HFpEF when EF is presevered, but preload reserve is impaired.

A primary drawback of using FF to characterize preload is that it requires an estimate of the dead-space or volume intercept of the PRSW relation, V_W_. Classically, this intercept is determined using preload variation, wherewith a plot of stroke work (SW) versus End-Diastolic Volume (EDV) yields slope (M_W_) and V_W_. Preload manipulation, however, is difficult and/or contraindicated in critically ill patients. Therefore, we developed a single beat method for determining the intercept, and in so doing, FF and M_W_ become more easily determined, single-beat measures.

## Mathematical Preliminaries

To better quantify factors that influence EF, consider a preload recruitable stroke-work (PRSW) model for heart function with SW linearly related to EDV as follows.

1 $$ {\rm{SW}}\,{\rm{ = }}\,{{\rm{M}}_{\rm{W}}}{\rm{*}}\,\left( {{\rm{EDV - }}{{\rm{V}}_{\rm{W}}}} \right) $$

where M_W_ represents the contractility of the heart and V_W_ represents the dead-space volume of the left ventricle (LV). Given that negative SW is unphysiological, V_W_ is, in essence, “dead-space” or the absolute minimum volume (AMV) of the LV. In this work, V_W_ is used as the PRSW intercept and as the actual, prescribed dead-space volume of the LV in the models herein. In contrast, AMV is intended to be more general and to represent the conceptual reality that the LVV has a lower limit that is non-zero when there is no preload. For PRSW, AMV is V_W_ because the preload metric, EDV-V_W_, is zero when EDV equals V_W_. If a different metric is used to define preload, e.g. consider EDP, then AMV would be the EDV when that measure is null (i.e., EDP = 0). Herein, AMV is also used as the single-beat estimate of the dead-space volume of the LV.

With AMV substituted for V_W_ and with SW given by the average power divided by heart rate (HR) (i.e., CO*MAP/HR, or equivalently, MAP*SV), then upon substitution and division by EDV, the PRSW relation becomes.

2 $$ {\rm{MAP}}\,{\rm{*}}\,{\rm{SV}}\,{\rm{/}}\,{\rm{EDV}}\,{\rm{ = }}\,{{\rm{M}}_{\rm{W}}}{\rm{*}}\,\left( {{\rm{EDV - AMV}}} \right)\,{\rm{/}}\,{\rm{EDV}} $$

Of course, SV/EDV is the EF. As for the volumes on the right-hand side of Eq. (2), the quantity EDV minus AMV is the “fill volume” (FV) or the volume that is added to the dead-space volume. Hence, FV = 0 appropriately represents a heart with “no filling”, (i.e., when FV = 0 the EDV is the dead-space volume). In so doing, let the filling fraction (FF) be the ratio of FV to EDV as defined by

3 $$ {\rm{FF}}\,{\rm{ = }}\,\left( {{\rm{EDV - AMV}}} \right){\rm{/EDV}} $$

Consequently, the PRSW relation becomes.

4 $$ {\rm{EF}}\,{\rm{ = }}\,{\rm{FF}}\,{\rm{*}}\,{{\rm{M}}_{\rm{W}}}{\rm{/}}\,{\rm{MAP}} $$

This expression for EF is, perhaps, the most concise representation for EF possible because it precisely states, with 3 separate factors, how EF depends on preload, contractility, and afterload. EF is: (1) directly proportional to FF, (2) directly proportional to M_W_, and (3) inversely proportional to MAP.

### Remark on Units

Note that EF and FF are unitless whereas M_W_ and MAP are typically reported in different units, i.e., M_W_ is reported with use of SI units as g/cm^2^ whereas conventionally MAP departs from SI and is reported in units of mmHg. Nevertheless, work per volume (i.e., M_W_) and pressure (i.e., MAP) are fundamentally the same unit. Given the fact that M_W_/MAP is a critical factor for EF, it is recommended that M_W_ and MAP both be reported in the same units for consistency. To do so, it is likely that M_W_ would be reported in mmHg because MAP will continue to be reported in mmHg. Alternatively, M_W_ can be reported as a percentage of MAP. The best units to use in a clinical setting is beyond the scope of this manuscript, and we use g/cm^2^ for M_W_ to be consistent with prior reports of M_W_.

## Clinical Relevance of FF for HFrEF

For a PRSW model, the maximum possible value for EF is FF because the lowest possible value for end systolic volume (ESV) is AMV (i.e., at AMV the cardiac muscle cells are so contracted that the heart cannot perform any SW). Practically, ESV is greater than AMV because the afterload is never zero. Based on data in the literature. EF is expected to be at least 10% less than FF. Furthermore, this upper limit for EF is critical for HFrEF. Toward this point, consider the data in Table 1 in Takeuchi, et al. [1] reporting V_W_/BSA and EDV/BSA for patients with EF > 50% (group 1, control group, mean EF = 0.66) and with EF < 50% (group 2, heart failure group, mean EF = 0.40). Comparing group 2 to group 1, V_W_ increases by 310% (19.1 ml/m^2^ to 59.3 ml/m^2^) whereas EDV increases 134% (95.1 ml/m^2^ to 127.5 ml/m^2^). Because V_W_ (i.e., AMV) increases nearly 3x more than EDV, FF is greatly reduced from 0.80 (group 1) to 0.53 (group 2). Relative to FF, EF is 14% less in group 1 and 13% less in group 2. This data is the basis for our statement that EF is expected to be at least 10% less than FF.

Moreover, the reduction in FF from 0.80 to 0.53 means the upper limit for EF is practically at the 0.5 threshold of HFrEF, even without considering contractility and afterload. In other words, a decrease in FF is extremely important because it lowers the ceiling for EF, and when the ceiling is at 0.53, an EF below 0.5 is inevitable. Of course, it is well recognized that a decrease in contractility (i.e., M_W_) reduces EF. Nevertheless, reduction in M_W_ needs to be reconsidered as the primary driver for a reduced EF because, for group 2 (i.e., low EF group), FF decreases by a larger percentage than M_W_ (i.e., 33% for FF and 21% for M_W_). Because EF is equally sensitive to both FF and M_W_, it follows that the EF reduction in group 2 is primarily due to FF reduction rather than M_W_ reduction, and to quantify each proportion, FF influence is 60% whereas M_W_ influence is 40%.

## Methods

In order to estimate the dead-space volume by way of a single-beat method (i.e., AMV), the relationship between the perimeter of the mid-wall (PMW) and left ventricular volume (LVV), and the relationship between PMW and wall-stress are key considerations. These two relationships can be estimated with single-beat methods, and then combined to yield AMV, if it is assumed that wall-stress goes to zero when LVV approaches AMV. The justification for this assumption is that the heart muscle cannot perform any work unless LVV exceeds AMV, and since wall-stress is the driving force for SW, wall-stress must be vanishing when LVV approaches AMV.

### Geometric Preliminaries

This analysis utilizes measurements of heart geometry in two conventional heart states, end systole (ES) and end diastole (ED), to predict the geometry in a third state, absolute minimum (AM). With ES, ED, and AM representing these states, the parameters in ED are: EDV, LVID_ED_, H_ED_, and PMW_ED_ (where LVID is LV internal diameter, and H is wall-thickness). Note that PMW is $\:\pi\:$ times the sum of LVID plus H.

In the ES state there is an additional important parameter that must be considered: end systolic-stress (ESS), which can be estimated from echocardiography measurements as described by Grossman, et al. [2] Let λ represent the stretch of the mid-wall chord relative to the AM state, wherewith λ_AM_ is unity, λ_ES_ is PMW_ES_ / PMW_AM_, and λ_ED_ is PMW_ED_ / PMW_AM_. Finally, the rate-corrected velocity of circumferential fiber shortening (V_CFC_) is the change in PMW from ED to ES (i.e., PMW_ED_ minus PMW_ES_) normalized for initial fiber length (by dividing by PMW_ED_) and rate-corrected (by dividing by the cube-root of the RR interval in seconds).

### Method to Estimate PMW_AM_ from ESS vs. λ_ES_

First, it is important to recognize that myocyte stress vs. stretch can be assumed to be linear when the contraction is static at the end of systole. Upon dividing the myofiber length by the zero-stress length, such a relation can be expressed with stress as a linear function of stretch with the x-intercept at 1 [3]. Second, λ_ES_ is an indicator of myocyte stretch at ES because, in the mid-wall region of the heart, myocyte orientation is relatively circumferential and myocyte stretch is relatively homogeneous despite inhomogeneous deformation [4]. With λ_ES_ approximating the myocyte stretch, the following linear relation can be constructed:

5 $$\:ESS={G}_{C}{M}_{N}\left({\lambda\:}_{ES}-1\right)$$

where M_N_ represents the slope when the contractility is normal and G_C_ represents a contractility gain. G_C_ is a positive number that: equals 1 for normal contractility, >1 for increased contractility, or < 1 for reduced contractility. For normal hearts (i.e., G_C_ = 1), λ_ES_ is approximately 1.12 [4] and ESS is approximately 60 g/cm^2^ [5]. Therefore, M_N_ can be approximated as 500 g/cm^2^.

G_C_ can be considered a function of Z_C_, the number of standard deviations that contractility is away from normal (i.e., Z_C_ is a Z-score for contractility with magnitude representing the number of standard deviations from mean and sign indicating below or above the mean). Let G_C_ be dependent on Z_C_ as follows:

6 $$\:{G}_{C}={C}_{1}{Z}_{C}+\sqrt{{\left({C}_{1}{Z}_{C}\right)}^{2}+1}$$

where, C_1_ is estimated to be about 0.2 (see appendix). As such, any contractility measure with a well-characterized normal distribution can be used for to get G_C_ from Z_C_. For example, let Z_C_ be quantified using the single-beat measure of contractility obtained from the V_CFC_ vs. ESS relation [6]:

7 $$\:{Z}_{C}=\frac{{V}_{CFC}+\left(\frac{0.0044{s}^{-1}c{m}^{2}}{g}\right)ESS-1.23\:{s}^{-1}}{0.06\:{s}^{-1}}$$

The mean-normal line (as obtained from 78 normal subjects [6]) can be obtained from this equation by setting Z_C_ = 0. The numerator is the vertical displacement from the mean-normal line, and the denominator is the vertical standard deviation of the distribution. Thus, the ratio of vertical displacement to vertical standard deviation is the number of standard deviations from the mean-normal line with positive sign indicating above the line and negative below.

With ESS measured and G_C_ calculated from Z_C_, the ESS vs. λ_ES_ relation yields a value for λ_ES_.

8 $$ {{\rm{\lambda }}_{{\rm{ES}}}}{\rm{ = }}\,{\rm{1}}\,{\rm{ + }}\,{\rm{ESS}}\,{\rm{/}}\,\left( {{{\rm{G}}_{\rm{C}}}{\rm{500 g/c}}{{\rm{m}}^{\rm{2}}}} \right) $$

Since λ_ES_ is the ratio of PMW_ES_ to PMW_AM_, a value for PMW_AM_ is obtained by.

9 $$ {\rm{PM}}{{\rm{W}}_{{\rm{AM}}}}{\rm{ = }}\,{\rm{PM}}{{\rm{W}}_{{\rm{ES}}}}{\rm{/}}\,\left( {{\rm{1 + }}\,{\rm{ESS}}\,{\rm{/}}\,\left( {{{\rm{G}}_{\rm{C}}}{\rm{500 g/c}}{{\rm{m}}^{\rm{2}}}} \right)\,} \right) $$

Provided that a single-beat measure is used to estimate G_C_, this is the single beat estimate for PMW_AM_ .

### Method to Estimate AMV from LVV vs. PMW

With an estimate for PMW_AM_, an estimate for AMV can be obtained from an approximation of the LVV vs. PMW relationship. For any axis-symmetric ellipsoidal shell, the volume enclosed depends on the cube of the cross-section perimeter, provided the axis dimension proportionally varies with the hoop dimension. Moreover, the proportion does not need to be equal, i.e., the axial shortening does not need to be equal to the hoop shortening; rather the proportionality factor becomes a coefficient. If PMW is the cross-sectional perimeter of a mid-wall shell, then a first-order approximation for LVV is

10 $$ {\rm{LVV}}\, = a{\rm{PM}}{{\rm{W}}^3}--b $$

where *b* is the volume of muscle within the mid-wall shell (i.e., as LVV goes to zero, PMW is not zero). The constants *a* and *b* depend on geometric features such as thickness-to-radius, axial-to-hoop dimension, and axial-to-hoop motion. For easy geometries, a and b can be calculated. For example, with LVV as a half sphere, then *a* is 0.00845 and *b* is the volume of wall between the mid-wall and inner-wall. Nevertheless, measurement of *LVV* and PMW for the ES and ED states allows *a* and *b* to be custom fit, for patient-specific heart size and motion without specifying all of the geometric factors. In particular

11 $$ a = \,\left( {{\rm{EDV}}{\mkern 1mu} - {\rm{ESV}}} \right)\,{\rm{/}}\,\left( {\,{{\left( {{\rm{PM}}{{\rm{W}}_{{\rm{ED}}}}} \right)}^{\rm{3}}} - {{\left( {{\rm{PM}}{{\rm{W}}_{{\rm{ES}}}}} \right)}^{\rm{3}}}} \right) $$

12 $$ \begin{array}{*{20}{l}}{b = - \left( {{\rm{ESV}}\,{\rm{*}}\,{{\left( {{\rm{PM}}{{\rm{W}}_{{\rm{ED}}}}} \right)}^{\rm{3}}} - {\rm{EDV}}\,{\rm{*}}\,{{\left( {{\rm{PM}}{{\rm{W}}_{{\rm{ES}}}}} \right)}^{\rm{3}}}} \right)\,{\rm{/}}\,}\\{\left( {\,{{\left( {{\rm{PM}}{{\rm{W}}_{{\rm{ED}}}}} \right)}^{\rm{3}}} - {{\left( {{\rm{PM}}{{\rm{W}}_{{\rm{ES}}}}} \right)}^{\rm{3}}}} \right)}\end{array} $$

With *a* and *b* obtained, a single-beat estimate for PMW_AM_ yields a single-beat estimate of AMV as follows:

13 $$ {\rm{AMV}}\, = a\,{\rm{PM}}{{\rm{W}}_{{\rm{AM}}}}^3 - b $$

### Method Verification In-Silico

To test the robustness of the proposed method to estimate AMV for a wide variety of heart geometries and contractile motions, the LV was modeled as a truncated prolate-ellipsoid shell with uniform thickness using R version 4.2.0. Uniform random sampling was used to generate 10,000 different hearts with variations in: LVID_AM_, H_AM_, truncation of the ellipsoid, ratio height to width, λ_ES_, and λ_ED_. LVID_AM_, the internal diameter of the fully contracted LV, was varied from 2.6 cm to 4.6 cm. H_AM_, the thickness of the LV wall in the fully contracted state, was varied from 1.0 cm to 2.0 cm. The ratio of height to width ranges from 1:1 (spherical) to 2:1. The truncation level of the ellipsoid, L, varied from 0 (half shell) to 0.375 (7/8 of an ellipsoid shell). Physiological correlations (e.g., between wall thickness and diameter) were not enforced and parameters were sampled independently. The ratio of stretch of the midwall chord at end diastole, λ_ED_, was varied from 1.24 to 1.36. The ratio of stretch of the midwall chord at end systole, λ_ES_, was varied from 1.06 to 1.18. R code is available upon reasonable request.

Upon inflating the shell with the volume of muscle assumed constant, the geometric shape of the model is constrained and the mid-wall chord and EDV can be determined as a function LVID or H. Because of the assumption of constant muscle volume, LVID and H are non-linearly related in an inverse, one-to-one manner. Starting at the fully contracted state, the model was inflated until the randomly assigned value of λ_ES_ was achieved allowing calculation of volumes and dimensions in the ES state. Subsequently, the model was further inflated until the randomly assigned value of λ_ED_ was achieved allowing for calculation of volumes and dimensions in the ED state.

**Fig. 1:**
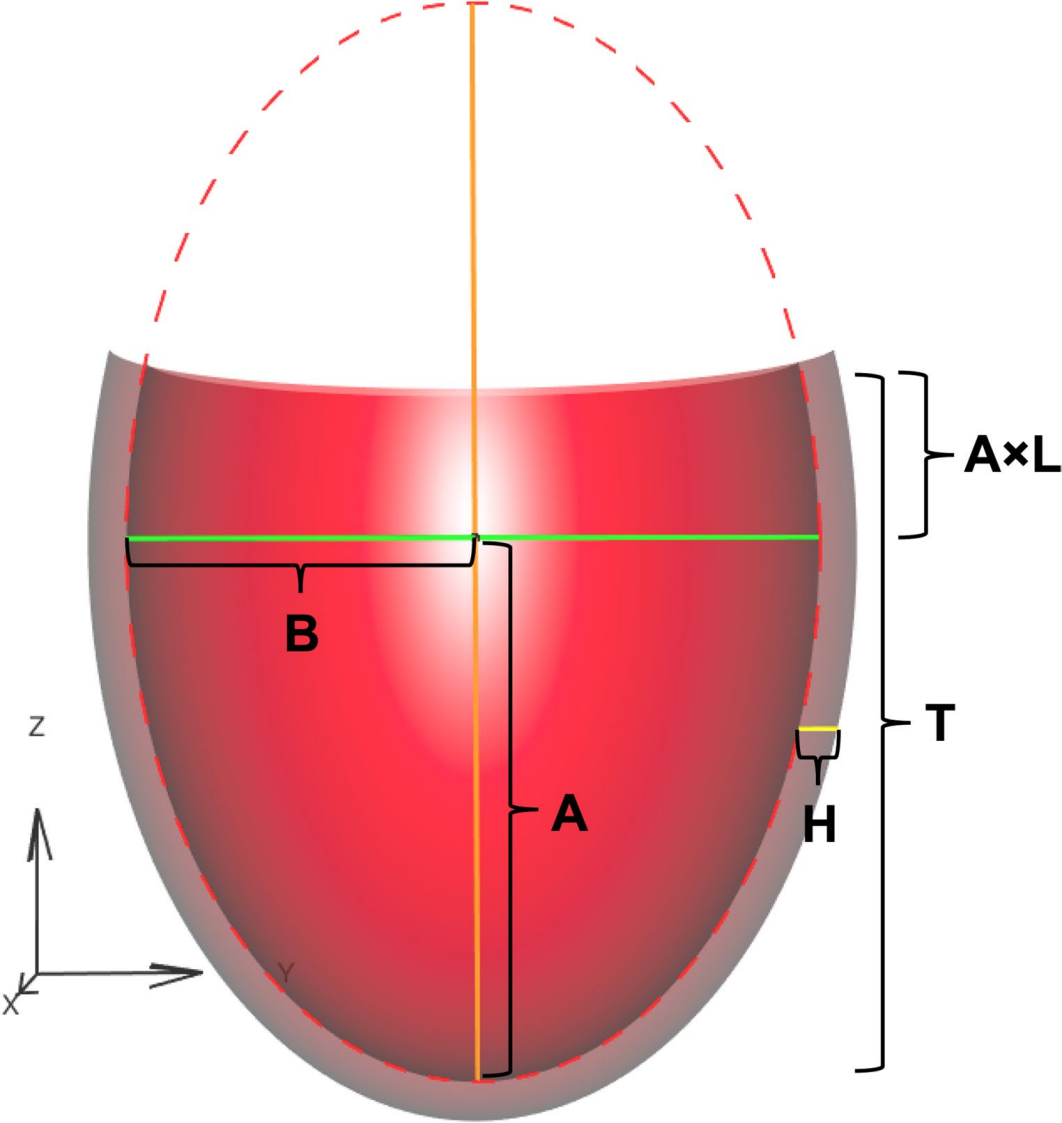
3-dimensional, truncated, axis-symmetric, prolate ellipsoid shell with uniform thickness. The shell is bisected in the x dimension for visualization purposes. **A**: major axis of the ellipse; **B**: minor axis of the ellipse; **H**: wall thickness of the shell; **L**: ratio of the long-axis height above the equator relative to the length of the ellipse below the equator; **T**: the total height of the truncated shell.

To calculate the volume of the left ventricle (LVV), the LV is assumed to be a truncated, prolate ellipsoid shell with a constant wall volume as depicted in Fig. 1. To define the ellipse of the internal cavity of the LV, consider an ellipse with major axis, *A*, and minor axis, *B* (i.e., ½ LVID). The ratio of the long-axis height above the equator relative to the length of the ellipse below the equator is represented by *L*. Thus the full length of the long axis of the truncated ellipse, *T*, can be calculated as a function of *A* and *L* according to the equation:

14 $$\:T=A(1+L)$$

For our purpose, *L* ranges from 0 (i.e., the equator of the ellipse is the level of truncation and *T = A*) to ¾ (i.e., the LV is 7/8 of a full prolate ellipsoid and *T = 1.75 A*); an *L* value of 1 would be a complete, not truncated, prolate ellipsoid wherewith *T = 2 A*. Thus we can define the internal volume of the full truncated ellipsoid shell, LVV, as:

15 $$\:LVV=\frac{2}{3}\pi\:A{B}^{2}\left(1+L+{L}^{2}-{L}^{3}\right)$$

Similarly, the volume of the LV myocardium, *V*_*M*_, is defined as:

16 $$\:{V}_{M}=\frac{2}{3}\pi\:AH\left(1+L+{L}^{2}-{L}^{3}\right)\left(H+2B\right)$$

For each modeled heart, we assume an incompressible myocardium (i.e., *V*_*M*_ is constant) and constant geometric shape (i.e., *L* is constant) over the entire cardiac cycle. Thus, combination of Eqs. 15, 16 and defined stretch ratios (λ_ES_, λ_ED_) enables calculation of the LVV and H throughout the cardiac cycle when the shape (i.e., *L* and the ratio of height to width) and dimensions (LVID and H) in any single state are known.

**Fig. 2:**
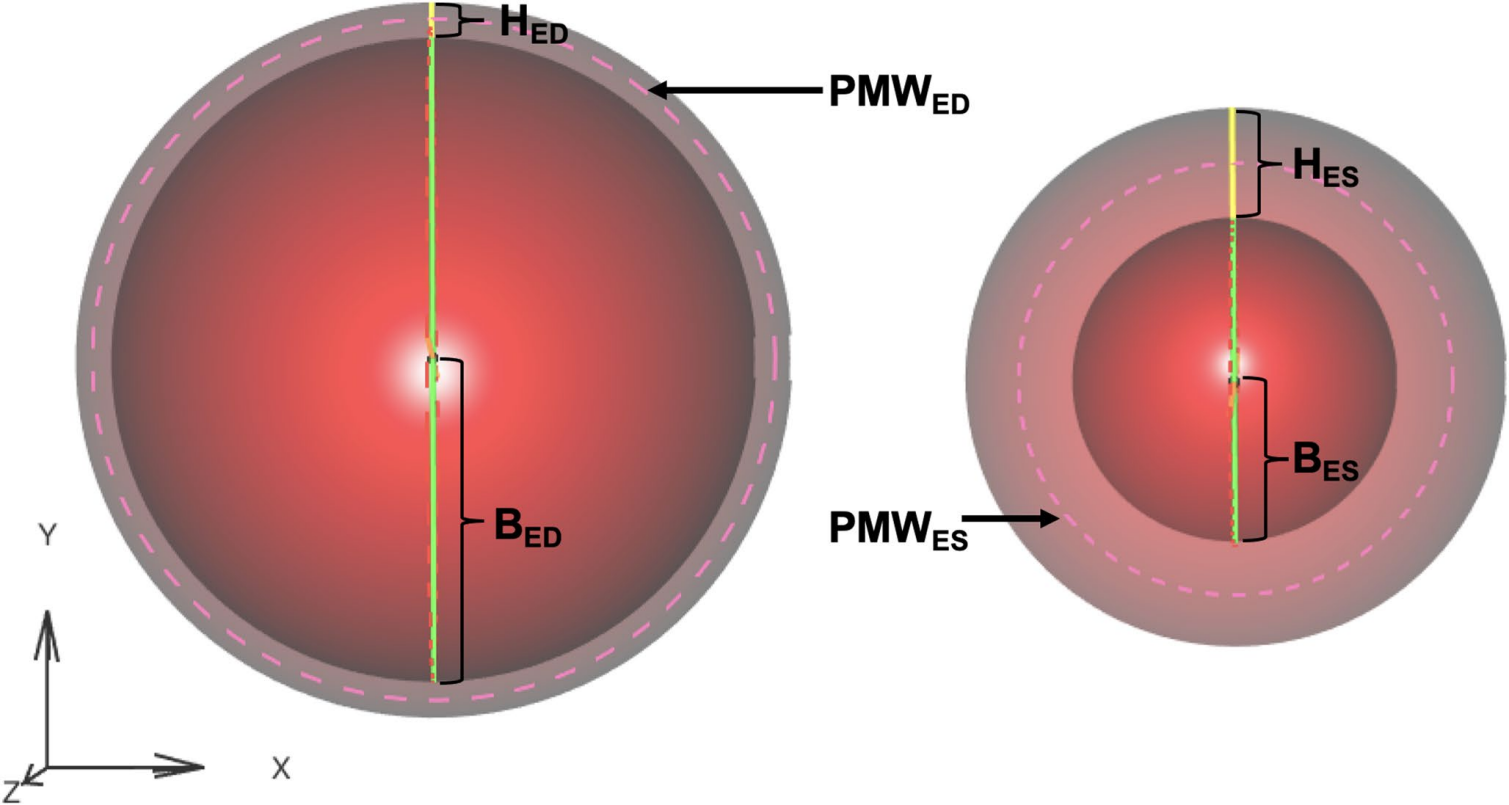
3-dimensional, truncated, axis-symmetric, prolate ellipsoid shell with uniform thickness. The shell is bisected in the z dimension at the equator for visualization purposes. The shell is shown in the end diastolic (ED) state (left) and end systolic (ES) state (right). The perimeter of the mid-wall chord (PMW) in each state is indicated by the pink dashed line. B_ED_: minor axis of the ellipse at end diastole; B_ES_: minor axis of the ellipse at end systole; H_ED_: wall thickness of the shell at end diastole; H_ES_: wall thickness of the shell at end systole; PMW_ED_: perimeter of the mid-wall chord at end diastole; PMW_ES_: perimeter of the mid-wall chord at end systole.

The PMW, shown in Fig. 2, is defined as:

17 $$\:PMW=\pi\:(2B+H)$$

## Results

**Fig. 3:**
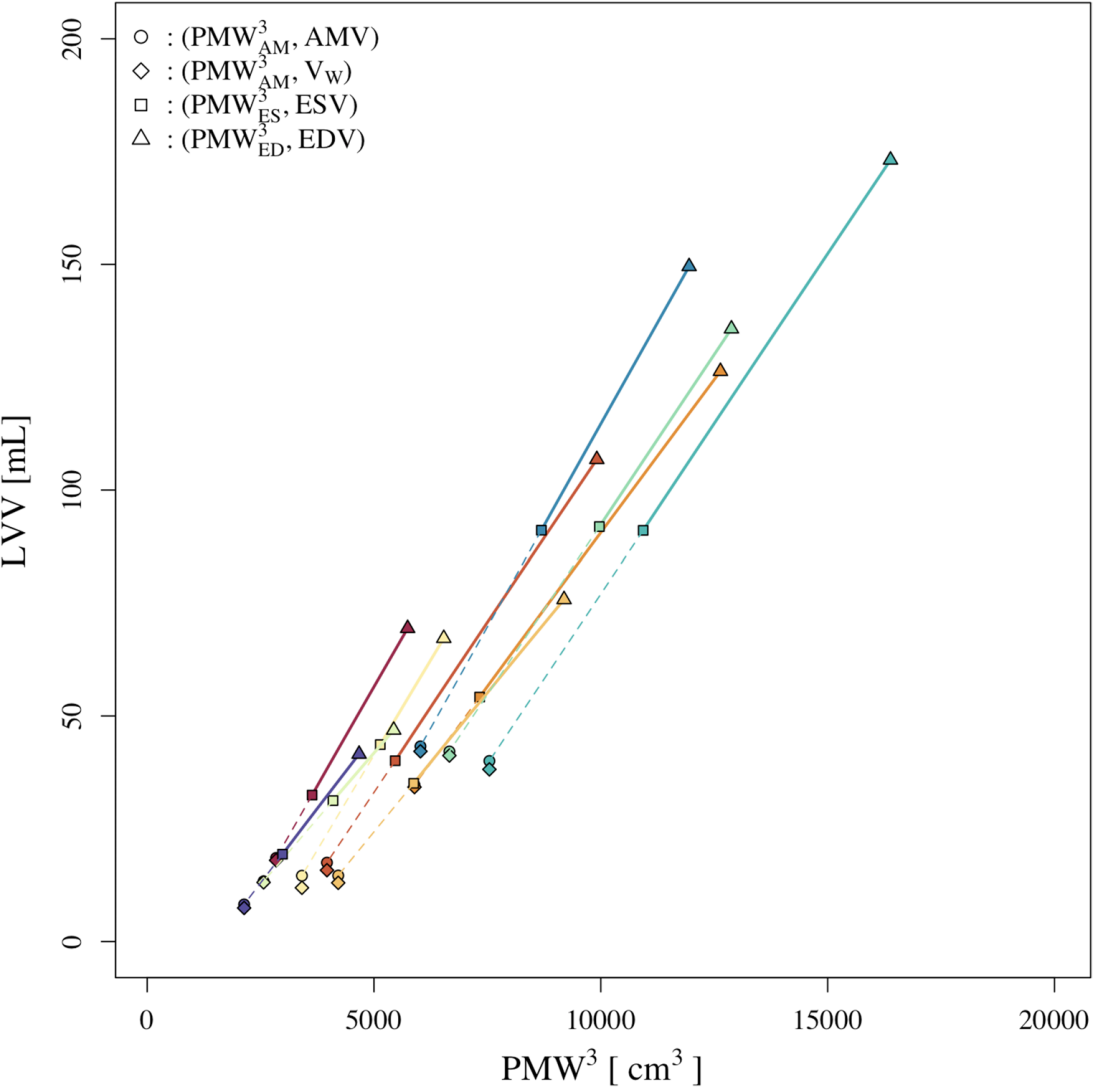
Perimeter of the mid-wall chord (PMW) cubed, vs. left ventricular volume (LVV) for 10 representative models. Each color represents one model. For each model, a solid line connects the end systolic state and end diastolic state; a dashed line connects the end systolic state to the predicted absolute minimum volume state. AMV: absolute minimum volume; PMW_ED_: perimeter of the mid-wall chord at end diastole; PMW_ES_: perimeter of the mid-wall chord at end systole; V_W_: dead space volume of the left ventricle.

For each model, the relationship of LVV vs. PMW was generated and tested for linearity of LVV relative to PMW^3^; In Fig. 3, 10 representative models are plotted to visualize this linear relationship of LVV relative to PMW^3^; the AMV^3^ is marked by a circle and the V_W_^3^ is marked with a rhombus for each of the 10 representative models.

**Fig. 4:**
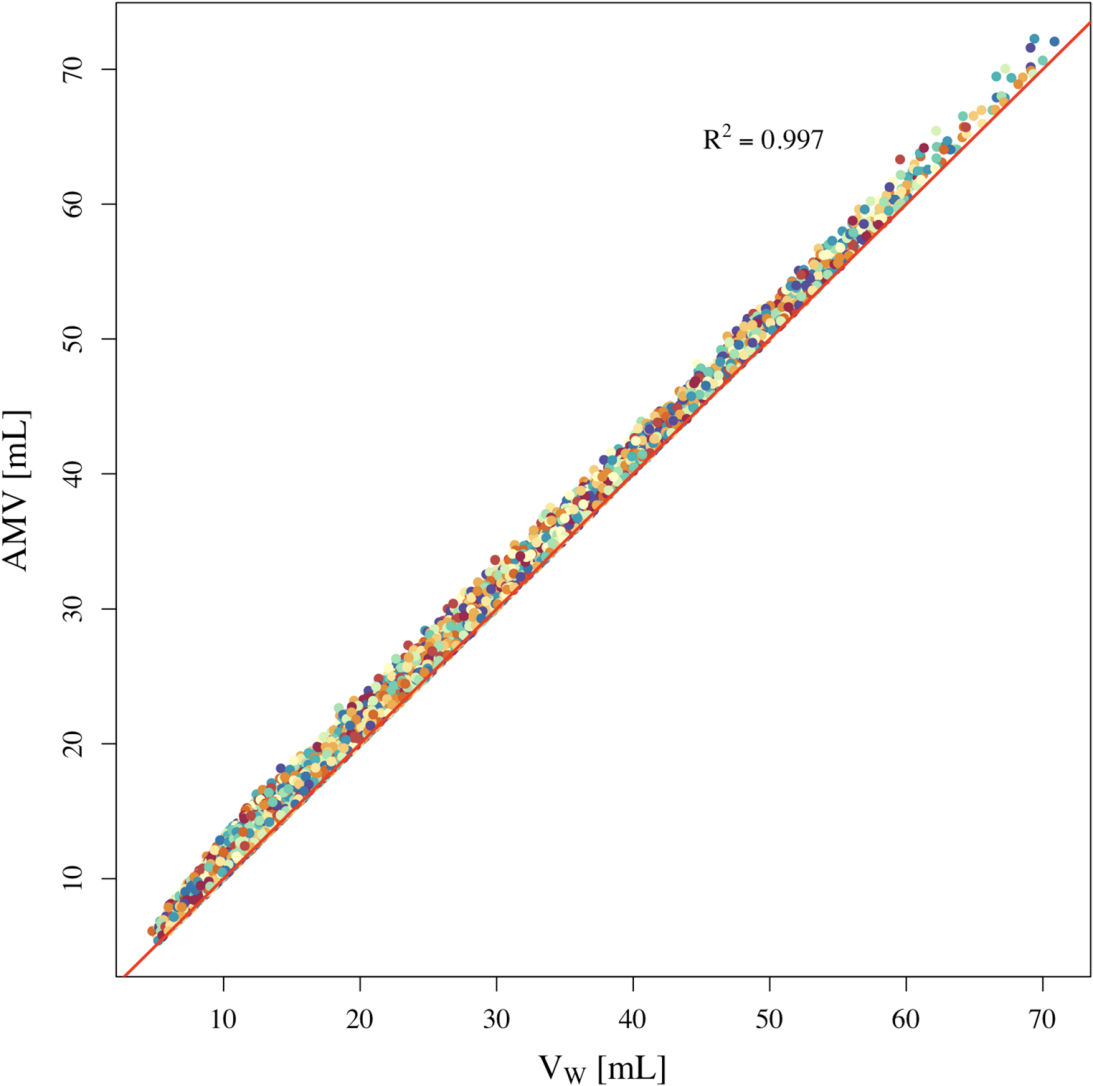
Dead space volume of the left ventricle (V_W_) vs. absolute minimum volume (AMV) 10,000 models. The redline represents a line of perfect fit where V_W_ is equal to AMV. AMV: absolute minimum volume; V_W_: dead space volume of the left ventricle.

Moreover, AMV was predicted using ES and ED states, as per Eqs. (10–12). This predicted AMV is compared to the V_W_ of each model as calculated based on the known dimensions in the fully contracted state. The V_W_ vs. AMV for each of the 10,000 models is plotted in Fig. 4.

## Discussion

The concept of FF is physiologically significant as it defines the upper limit for EF, given that EF cannot exceed FF without violating the PRSW model. Moreover, regardless of the PRSW model, there is a physical dead-space volume for the LV, so EF = 1 is unrealistic because the actual upper-limit for SV is EDV minus the dead-space volume. Furthermore, a linear dependence between EF and FF, as inspired by PRSW, may be useful independently and should be easy to validate with in-vivo and in-silico studies. Toward this end, note that EF is known to have has a non-linear dependence on EDV, wherewith EF depends significantly on EDV when EDV is low and becomes insensitive when EDV is high [7]. FF has a similar dependence on EDV, and hence, a linear dependence of EF on FF is likely.

Reduction of FF in HFrEF patients highlights an important limitation on EF. Using previously published data [1] it is shown that reduction in FF is the dominant contributor to the decreased EF observed in HFrEF. This challenges the prevailing emphasis on contractility deicits in HfrEF because reduced contractility is only 40% of EF reduction whereas reduced FF is 60%. This gives rise to a treatment dilemma because increased preload is needed to increase FF, yet more preload leads to pulmonary congestion. Perhaps, the only option is to treat the root cause of the FF reduction, i.e. treat the 3x increase in dead-space volume that accompanies dilatation. Such structural heart treatments to reduce dilatation are theoretically most promising.

A key contribution of this study is the development of a single-beat method for estimating the dead-space or absolute minimum volume (AMV), which previously required preload variation and extrapolation [8]—procedures that are impractical or contraindicated in critically ill patients. The single-beat method provides an estimate of AMV and consequently enables FF calculation without invasive or multi-beat measurements. In-silico modeling results demonstrate a strong correlation between the estimated AMV and the true dead-space volume (V_W_) across 10,000 simulated heart models, with deviations confined to expected physiological variability. This suggests that the proposed single-beat method for estimating dead-space volume of the LV is broadly generalizable and accurate across diverse functional states.

This study establishes FF as a physiologically and clinically significant metric for characterizing preload and its influence on EF. The proposed single-beat method for estimating AMV represents a substantial methodological advancement, enabling non-invasive, real-time assessment of FF. Potential clinical applications include integration of FF into cardiac imaging post-processing software and inclusion of FF in AI-driven functional assessment of cardiac health. These findings enhance understanding of the determinants of EF and have the potential to reshape diagnostic and therapeutic strategies for heart failure, particularly HFrEF.

Recent work, such as that of Lababidi et al., has sought to address diagnostic gaps through an algorithmic approach to classifying diastolic dysfunction with a framework that optimizes categorical assessment using binary thresholds and discrete decision steps [9]. Given that FF provides a continuous descriptor of diastolic filling dynamics, it may serve to augment current algorithmic approaches by offering a physiologically continuous variable capable of resolving discordant or borderline cases within stepwise frameworks. Additionally, incorporation of FF into machine-learning models that combine structural, strain-based, and Doppler variables may increase predictive accuracy. Future studies are needed to both evaluate inclusion of FF into existing diagnostic algorithms as well as correlate FF values with the degree of dysfunction clinically.

### Limitations

A PRSW model is the basis for this work, and deviations from this model would likely lead to EF being non-linearly dependent on FF. Moreover, FF requires estimate of the dead-space volume and a PRSW model provides that metric. A linear end-systolic pressure-volume relationship (ESPVR) model also provides an estimate of the dead-space, however, such a model does not have an EF factor (i.e., it only depends on ES state), so it could not be used to parameterize EF.

The single-beat estimate method is based on linearity of the end-systolic stress versus end-systolic stretch of myocytes and linearity of LVV versus PMW^3^. For ellipsoidal shells that span the entire range of heart sizes and shapes, it is shown that the method is accurate, yet validation is needed in more accurate in-silico models, animal models and, ultimately, human subjects.

The relationship for how Z_C_ maps to G_C_ (and the choice of 0.2 for C_1_) is a gross approximation based on combining results from different published studies. A purposely designed study is likely to provide a better estimate.

The in-silico model provides valuable insights and mathematical support for the proposed single-beat method to estimate AMV and FF, albeit subject to certain limitations inherent to computational modeling and presently not validated by independent groups. These limitations must be acknowledged to contextualize the findings and guide future work. First, the model operates on assumptions about LV geometry and biomechanics, such as uniform wall thickness, axisymmetric ellipsoidal shape, and linear relationships between certain parameters (e.g., wall stress and stretch at end-systole). These simplifications do not fully capture the complexity and variability of real-world cardiac structures and dynamics [10]. Although the model incorporates a wide range of parameters to simulate diverse heart geometries and motions, it may not account for all potential physiological variations. For example, regional heterogeneity in myocardial contractility, stiffness, and deformation—often seen in patients with ischemic heart disease or myocardial fibrosis—is not accounted for in this model. Furthermore, the model captures static states of the heart (e.g., ES, ED, and AMV) but does not account for dynamic processes such as diastolic filling patterns, myocardial relaxation, or temporal variations in contractile function. It is reasonable that large deviations from normal physiology could influence the real-world applicability of FF measurements. Although the relationships between parameters are internally consistent and mathematically robust, their applicability to real-world patient data remains to be externally validated through clinical studies.

## Figures and Tables

**Fig. 5:**
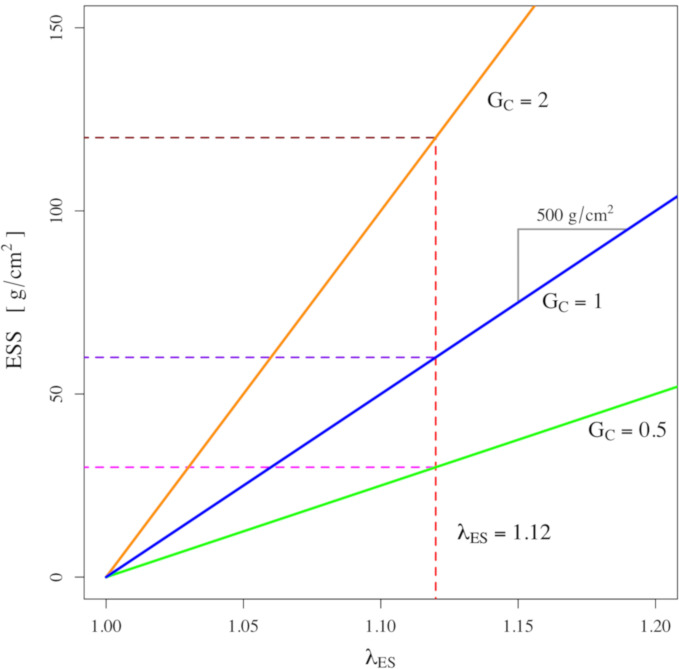
Stretch of the mid-wall chord (λ), vs. end systolic stress (ESS). Representative lines showing reduced contractility (green line, G_C_= 1/2), normal contractility (blue line, G_C_= 1), and increased contractility (orange line, G_C_= 2) are shown. G_C_: contractility gain; ESS: end systolic stress; λ_ES_: stretch of the mid-wall chord at end systole

**Fig. 6:**
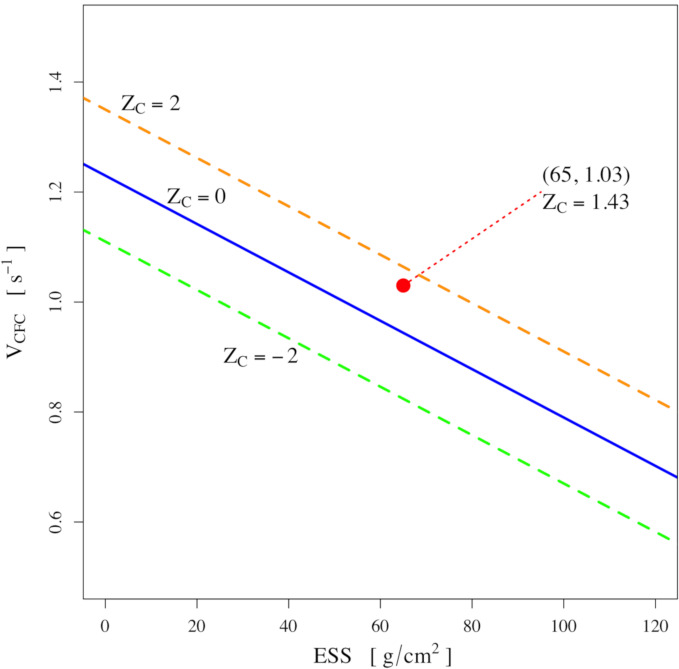
End systolic stress (ESS) vs. rate corrected, velocity of circumferential fiber shortening (V_CFC_). Representative lines showing a decreased contractile state (green line, Z_C_= -2), the mean-normal line (blue line, Z_C_= 0), an increased contractile state (orange line, Z_C_= 2), and a hypothetical patient (red circle, Z_C_ = 1.43) associated with the point (ESS = 65 g/cm^2^, V_CFC_ = 1.03 s^− 1^) are shown. ESS: end systolic stress; V_CFC_: velocity of circumferential fiber shortening; Z_C_: Z score of the contractility gain

**Fig. 7:**
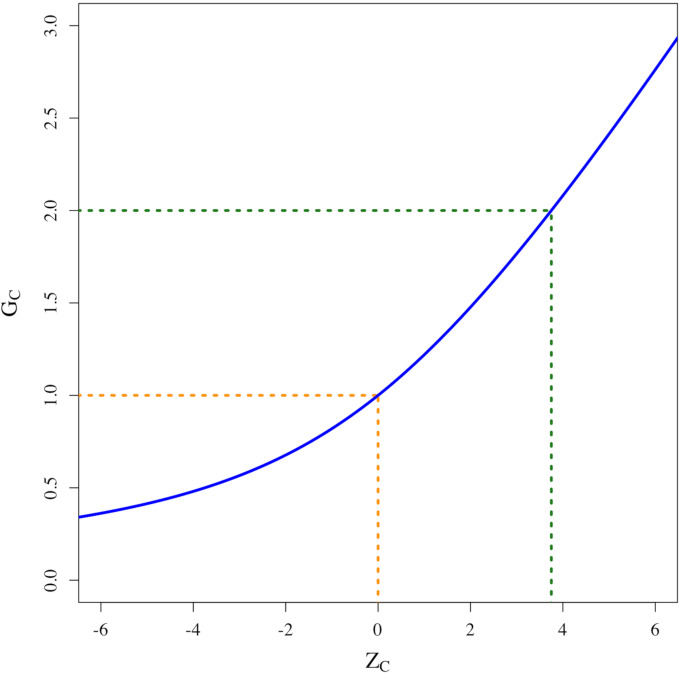
The relationship of Z_C_ vs. G_C_ as described in Eq. 15 with C_1_ = 0.2 (blue line). The intercept of this relationship with a state of normal contractility (Z_C_ =0, G_C_ = 1) is shown by the orange lines. The intercept of this relationship with a state of increased contractility (Z_C_ = 3.75, G_C_ = 2) is shown by the green lines. G_C_: contractility gain; Z_C_: Z score of the contractility gain.

## Data Availability

Not applicable.

## Appendix

Toward estimating M_N_, note that for normal hearts, λ_ES_ is approximately 1.12 [4] and ESS is normally 60 g/cm^2^ [5]. Hence, let M_N_ be approximated as 500 g/cm^2^.

Fig. 5 displays such a relation for normal contractility and with doubling and halving the slope (i.e., G_C_ equal to 1, 2, and 0.5, respectively).

As for determining G_C_, consider the patient’s deviation from the V_CFC_ vs. ESS relationship obtained from 68 normal subjects [6]. For a data point (ESS, V_CFC_) that is above the mean-normal line, a patient has increased contractility, and likewise for below the line, decreased contractility. To further quantify contractility relative to normal, let Z_C_ be a Z score for contractility with a sign indicating whether the patient’s state (ESS, V_CFC_) is above the line (positive) or below (negative). Moreover, the magnitude of Z_C_ represents the number of standard deviations away from the line. In so doing, Z_C_ is given by

18 $$\:{Z}_{C}=\frac{{V}_{CFC}+\left(\frac{0.0044{s}^{-1}c{m}^{2}}{g}\right)ESS-1.23\:{s}^{-1}}{0.06}$$

Fig. 6 plots the mean-normal line (solid) and dashed lines for Z_C_ = +/- 2. Also shown is a hypothetical patient associated with the point (ESS = 65 g/cm^2^, V_CFC_ = 1.03 s^− 1^), a contractile state with Z_C_ = 1.43.

To estimate how G_C_ relates to Z_C_, first consider the theoretical bounds with:

1. G_C_ = 1 when Z_C_ = 0.
2. G_C_ goes to zero as Z_C_ goes negative.
3. G_C_ increases proportionally to increasing Z_C_.

One such function with the proper theoretical behavior is:

19 $$\:{G}_{C}={C}_{1}{Z}_{C}+\sqrt{{\left({C}_{1}{Z}_{C}\right)}^{2}+1}$$

wherein C_1_ is a unitless fitting constant. This relation is shown in Fig. 7 (with C_1_ = 0.2) and it is invertible with

20 $$\:{Z}_{C}=\frac{\left({G}_{C}-{G}_{C}^{-1}\right)}{2{C}_{1}}$$

A study that measures G_C_ and Z_C_ simultaneously would be needed to precisely determine C_1_ and/or revise the functional relationship between G_C_ and Z_C_. Nevertheless, with data from separate studies, an estimate for C_1_ can be made. Toward this end, consider the dobutamine data points in Colan et al. [6] and note that these points are about 3–4 standard deviations above normal, i.e., with inotropic stimulation, Z_C_ is in the range of 3–4. As for G_C_, note that inotropic stimulation can nearly double the myocyte stress at a given stretch [3]. To estimate C_1_, note that C_1_ = 2.5 if Z_C_=3 when G_C_=2, and C_1_ = 1.875 if Z_C_=4 when G_C_=2. Hence, a first-order approximation is C_1_ = 0.2.
